# Stereo Imaging Using Hardwired Self-Organizing Object Segmentation

**DOI:** 10.3390/s20205833

**Published:** 2020-10-15

**Authors:** Ching-Han Chen, Guan-Wei Lan, Ching-Yi Chen, Yen-Hsiang Huang

**Affiliations:** 1 Department of Computer Science and Information Engineering, National Central University, Taoyuan 32001, Taiwan; pierre.miat@gmail.com; 2 Department of Information and Telecommunications Engineering, Ming Chuan University, Taoyuan 333321, Taiwan; chingyi@mail.mcu.edu.tw; 3 National Chung-Shan Institute of Science and Technology, Taoyuan 32546, Taiwan; wantai943@hotmail.com

**Keywords:** object segmentation, SOM, stereo vision

## Abstract

Stereo vision utilizes two cameras to acquire two respective images, and then determines the depth map by calculating the disparity between two images. In general, object segmentation and stereo matching are some of the important technologies that are often used in establishing stereo vision systems. In this study, we implement a highly efficient self-organizing map (SOM) neural network hardware accelerator as unsupervised color segmentation for real-time stereo imaging. The stereo imaging system is established by pipelined, hierarchical architecture, which includes an SOM neural network module, a connected component labeling module, and a sum-of-absolute-difference-based stereo matching module. The experiment is conducted on a hardware resources-constrained embedded system. The performance of stereo imaging system is able to achieve 13.8 frames per second of 640 × 480 resolution color images.

## 1. Introduction

Stereo vision is an imaging technique developed on the basis of biological vision principles. It involves using two cameras to simultaneously capture two images, comparing the images to find the objects that match, and estimating the distance between the objects and the cameras using the disparity of the objects within the two images. Therefore, image segmentation and stereo matching are often two important technologies for developing stereo vision systems. First, the image segmentation technique is used to separate the objects in the two images and identify the corresponding points of the same objects within both images for further matching. Then, the principle of stereo matching is used to acquire the depth of the objects. 

The purpose of image segmentation is to partition an image into several regions or objects that do not overlap and that have similar characteristics. Image segmentation methods can be divided into the following types: the thresholding method, edge-based method, region-based method, watershed method, clustering-based method, and neural-network-based method. The thresholding method [1,2] generates threshold values by analyzing the gray-level histogram of full or partial images and segments the objects within the images by clustering all of the pixels of these images. Of the numerous commonly used segmentation techniques, this method has been widely well-received because of its simple implementation procedures, robustness, and accuracy [3]. Even in recent years, academia has continued to develop novel thresholding approaches or improve existing ones [4,5,6,7,8]. The edge-based method [9] measures or extracts region boundaries by detecting the edges of images to identify objects in these images; this method has the advantage of low complexity, but it can also be easily affected by noise. The region-based method [10,11] utilizes the homogeneity of pixel features (e.g., gray scale, color, or texture) in the same region as the criterion of segmentation, the purpose of which is to partition an image into regions with distinct characteristics. The watershed method-based algorithm [12,13] segments images by considering an image to be a topographic map and utilizing variations in flood water heights and watershed lines.

Segmentation methods that do not use spatial information to group pixels into regions are often called clustering techniques. Clustering is a useful unsupervised data mining technique that partitions the input space into K regions depending on a similarity/dissimilarity metric. The clustering-based methods use measures of intraclass and interclass homogeneity to determine the optimal segmentation. The simplest clustering approach commonly employs well-known techniques such as K-means [14], fuzzy c-means [15], and probabilistic extension using the Gaussian mixture model and expectation–maximization algorithm [16]. This type of image segmentation is widely used due to the simplicity of understanding and accurate segmentation results.

Artificial neural networks (ANNs) are well known for their excellent performance in classification and function approximation and have been used with success in digital image processing. Neural network-based segmentation methods rely on processing small regions of an image using an ANN or a set of different ANNs. After such processing, the decision-making method marks the regions of an image according to the categories recognized by the neural network. A self-organizing map (SOM) is a type of ANN designed specially to address such problems [17]. The use of a SOM in image segmentation is well reported in the literature [18,19].

Because the development of stereo vision systems involves complex algorithms, powerful CPU resources and high-performance computational platforms are typically required. Thus, the performance of embedded systems used for stereo vision processing is often constrained by cost and technical limitations, which render it difficult to achieve real-time processing. Performing image segmentation solely using software is simply a computationally intensive task [20] and is therefore impractical for developing real-time embedded systems. However, several studies involving the hardware implementation of SOM have been conducted [20,21,22]; for example, in [21], Porrmann, Ruping, and Ruckert applied a scalable parallel architecture to understand SOM digital hardware, which features a classification rate of 250,000 vectors per second. In [22], the researchers designed a modular SOM systolic architecture that can classify data vectors with thousands of elements in real time. The architecture is described as a soft intellectual property core in synthesizable VHSIC hardware description language. Observing that a SOM architecture can be easily converted into parallel processing units, Kurdthongmee [20] proposed a novel Kohonen SOM-based architecture that involves using field programmable gate array (FPGA) chips. This architecture was developed based on unsigned integer arithmetic in all operations and performs adequately in terms of image quality, frame rate throughput, and FPGA chip resource utilization.

Building on this previous scholarship, the present study proposed a SOM-based object segmentation method that can be applied to stereo vision systems and employed FPGA techniques to develop a hardware accelerator that performs real-time and rapid object segmentation. We also designed a pipeline controller to control the operation of three modules (i.e., a SOM neural network module, connected component labeling [CCL] module, and stereo matching module) to effectively improve overall system performance and achieve the requirements of real-time processing and high efficiency. 

The remainder of this paper consists of five sections: Section II introduces relevant stereo vision techniques, Section III elaborates on the methods for designing the hardware of an embedded stereo vision system, Section IV presents the experiment results and compares system performance, and Section V provides a summary of the study findings.

## 2. Materials and Methods

### 2.1. Stereo Vision Algorithms

**Figure 1:**
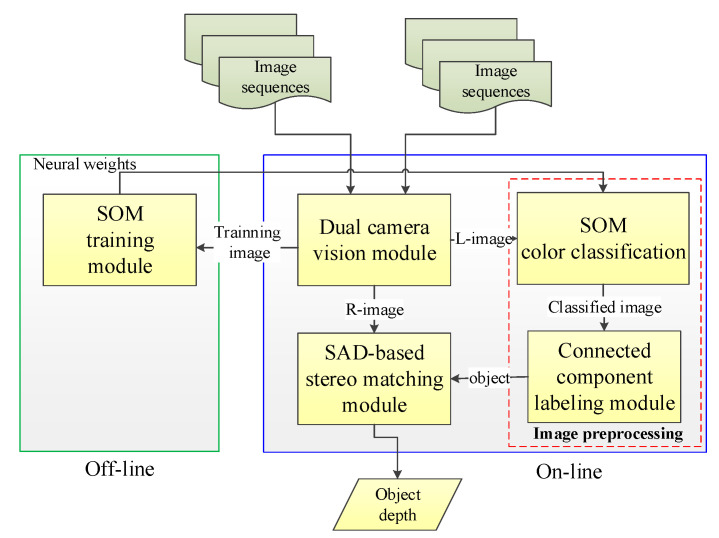
System structure of the proposed embedded stereo system.

Figure 1 presents the stereo vision system architecture proposed in this study. The dual camera vision module captures left and right images and sends the left images to the image preprocessing module for classification and CCL. Stereo matching is performed on the obtained objects and right images to estimate the similarity and disparity of the same object between two images. A lookup table (LUT) is then utilized to generate depth values.

#### 2.1.1. Self-Organized Map

A SOM [23] is a neural network based on competitive learning; that is, the neurons of the output layer compete with each other to reach an activated opportunity. In most competitive learning neural networks, the winner is selected through a competitive phase and the weight vector of the winner is adjusted in a rewarding phase. However, in a SOM, both the winning neuron and its neighboring neurons have a chance to learn after the competitive phase. The conventional SOM learning algorithm can be explained using the following steps:

(1) Initialize the weight vectors of the M × N neurons
(2) Repeat until convergence
  (a) Select the next input vector *x_i_* from the data set:
    (i) Find the unit Wj* that best matches the input vector *x_i_*
      (1) $$J^{*}=\underset{j}{argmin}∥x_{i}-W_{j}∥,\;j=1,\ldots ,M*N$$
    (ii) Update the weights of the winner Wj* and its neighboring neurons *W_k_*
    (2) $$JW_{k}=W_{k}+\eta \left(t\right)\cdot h_{j*k}\left(t\right)\cdot \left(x_{i}-W_{k}\right)$$
    where *h_j∗k_*(*t*) is the Gaussian neighborhood function given as
    (3) $$h_{j*k}\left(t\right)=\exp\left(-\frac{d_{j*k}^{2}}{2\sigma {\left(t\right)}^{2}}\right)$$
  (b) Select the next input vector *x_i_* from the data set:
    (4) $$\eta \left(t\right)=\eta_{0}\exp\left(-\frac{t}{\tau_{1}}\right)$$
  (c) Decrease neighborhood size *σ(t)* that defines the topological neighborhoods:
    (5) $$\sigma \left(t\right)=\sigma_{0}\exp\left(-\frac{t}{\tau_{2}}\right)$$

#### 2.1.2. Connected Component Labeling

CCL is a task that detects connected regions in input data, and which can be applied for pattern recognition and image segmentation. A CCL module scans an image and groups its pixels into components based on pixel connectivity. All pixels in a connected component share similar pixel intensity values and are in some way connected with each other. Once all groups have been determined, each pixel is labeled with a gray level or a color according to the component to which it was assigned. 

#### 2.1.3. Stereo Matching

The sum of absolute difference (SAD) technique [24] is the most common matching criterion in stereo matching algorithms because of its low complexity, excellent performance, and ease of hardware implementation. The technique computes the intensity differences for each center pixel (*i,j*) in a window *W*(*x*,*y*) as follows:

(6) $$\mathrm{SAD}=\sum_{\left(i,j\right)\in W}\left|I_{L}\left(i,j\right)-I_{R}\left(i,j+d\right)\right|,$$

where IL and IR denote the left and right image pixel intensity functions, respectively, and *W*(*x*,*y*) is the square window that surrounds the location (*x*,*y*) of a pixel.

**Figure 2:**
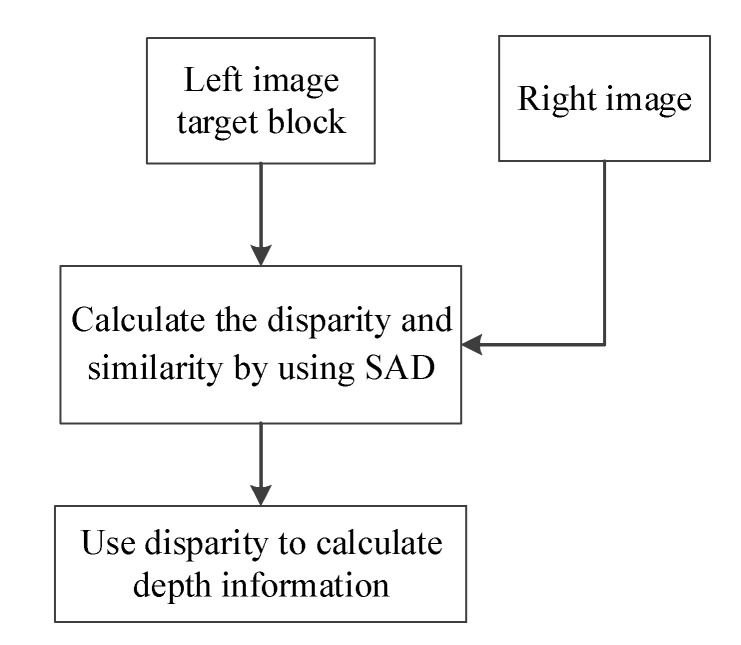
Stereo matching flowchart.

Figure 2 illustrates the process of stereo matching. First, a target block is selected from a left image as a template, which is then gradually moved along a baseline in the right image to compare both images and locate the block that best resembles the target block from the left image. Stereo matching is then conducted to calculate disparity values and determine the level of similarity between both blocks. Finally, an LUT or formula is used to obtain depth information about the objects in the images.

### 2.2. Hardware Architecture of the Embedded Stereo Vision System

#### 2.2.1. Dual Camera Vision Module

**Figure 3:**
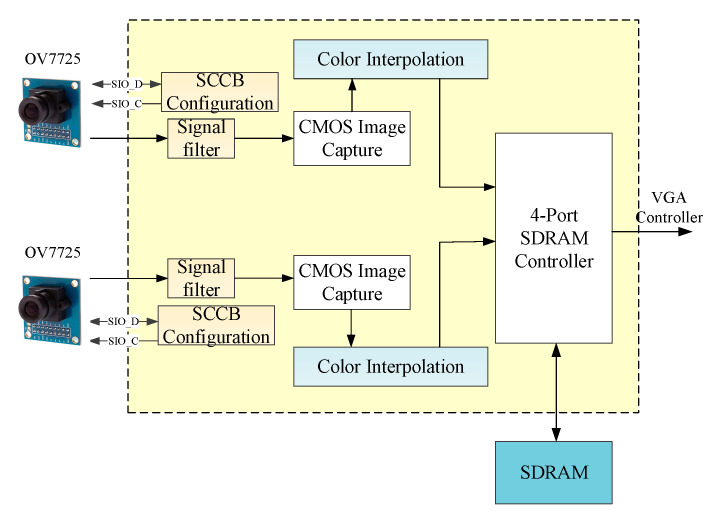
Dual camera vision module.

**Figure 4:**
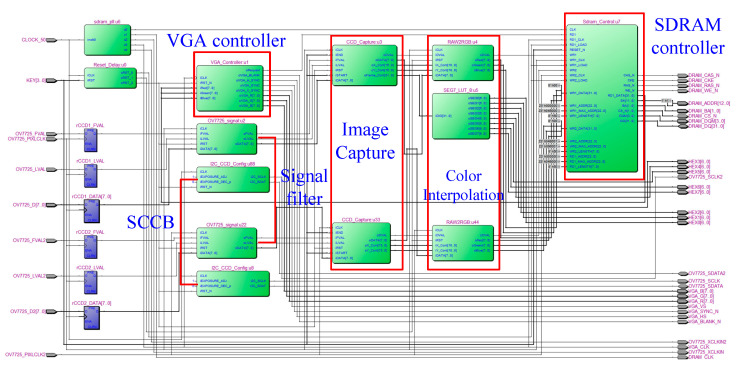
RTL-schematic diagram of the dual camera vision module.

Figure 3 shows the hardware architecture of the dual camera vision module. A signal filter removes excess noise, and a serial camera control bus module performs the initial setting of the complementary metal-oxide-semiconductor sensors on both sides. After receiving the input image data, the dual camera vision module then sends the data to the color interpolation module to generate RGB color-based image data, which is finally stored in a synchronous dynamic random-access memory (SDRAM) cell. The RTL schematic view of the dual camera vision module is presented in Figure 4.

#### 2.2.2. Self-Organized-Map-Based Image Segmentation Module

**Figure 5:**
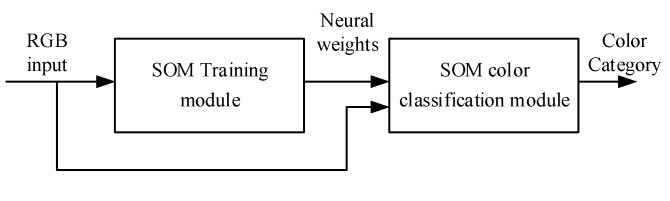
SOM-based image segmentation module.

The SOM-based image segmentation module contains two submodules, namely an SOM training module and an SOM color classification module (Figure 5). Notably, only the data of the first image is input into the SOM training module to train the color segmentation module; the data of the remaining images are input directly into the SOM color classification module to execute various pixel classification tasks.

##### SOM Training Module

**Figure 6:**
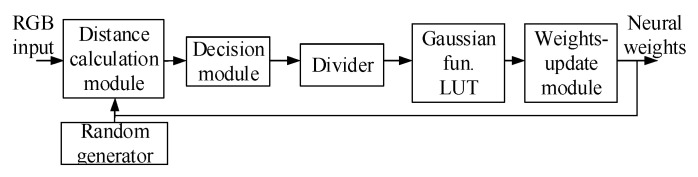
System architecture of the SOM training module.

**Figure 7:**
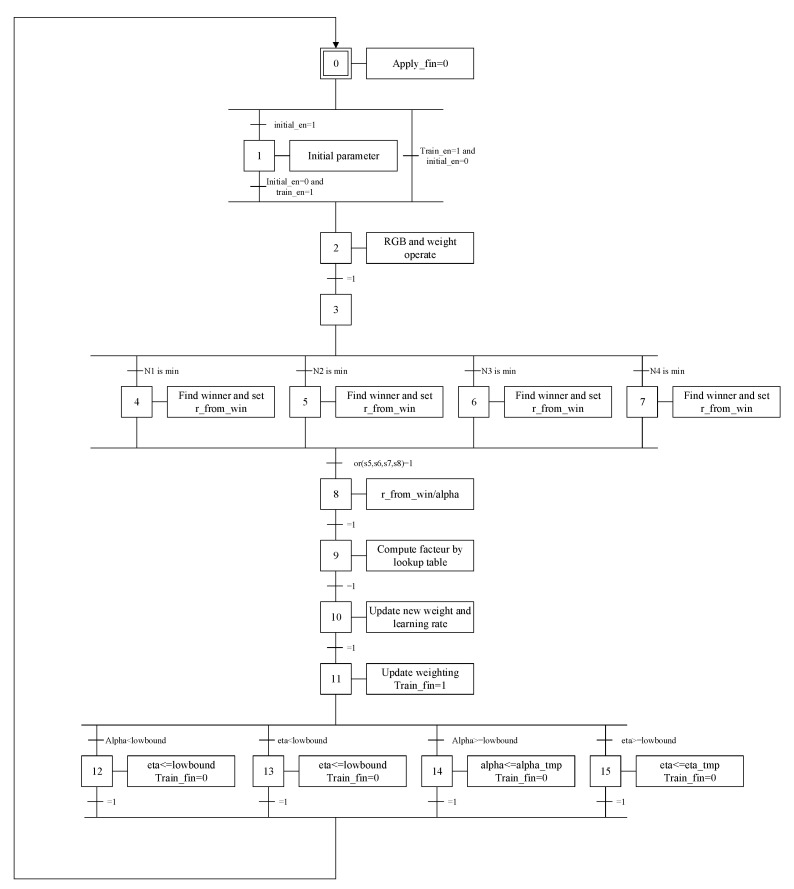
Grafcet discrete-event model of the SOM training module.

Figure 6 displays the system architecture of the SOM training module, wherein a random generator generates initial weights for the SOM. The distance calculation module and decision module are then used to calculate input vectors and the distance of weights for all output neurons, thereby determining the winner neuron. The weights-update module adjusts the bonding values of the winner neuron and its neighboring neurons. Subsequently, a divider provides the computational power for the weights-update module. Figure 7 presents the Grafcet discrete-event model [25] of the SOM training module.

##### Random Generator

**Figure 8:**
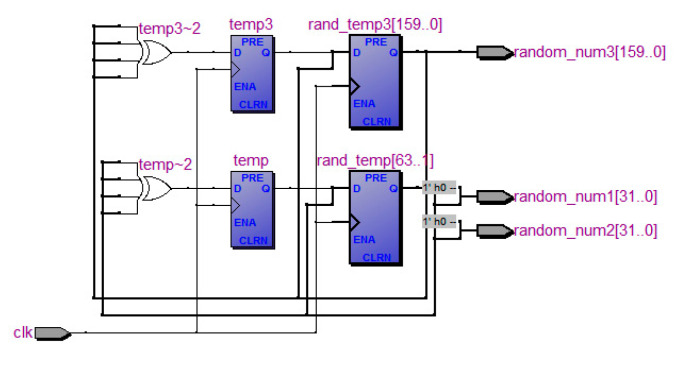
RTL-schematic diagram of the random generator.

During the training phase of a SOM, the initial weights of all neurons are random. Thus, a circuit module that generates random numbers must be designed. Random number generators using linear feedback shift registers (LFSRs) were developed in ref. [26]. Because the state of a register is often limited, the process of random number generation is a repeated cycle; however, the use of primitive polynomials enables the LFSRs to generate random sequences with extended cycle periods. LFSRs have been used to generate pseudo-random binary sequences, which are then used to derive the required random sequences. Figure 8 provides an illustration of the random generator for the SOM training module adopted in the present study, which was designed using LFSRs.

##### Lookup Table of Gaussian Function

In the SOM algorithm, a winner neuron stimulates its neighboring neurons through lateral connections. The level of stimulation is related to the distance between these lateral connections. Specifically, neurons at a shorter distance from the winner neuron are stimulated to a higher level, whereas neurons at a longer distance from the winner neuron are stimulated to a lower level. The Gaussian function employed in this study is expressed as (7):

(7) $$h_{j*k}=\exp\left(-\frac{d_{j*k}^{2}}{\alpha }\right)$$

##### SOM Color Classification Module

**Figure 9:**
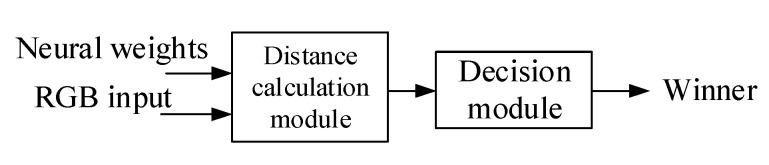
System architecture of the SOM color classification module.

**Figure 10:**
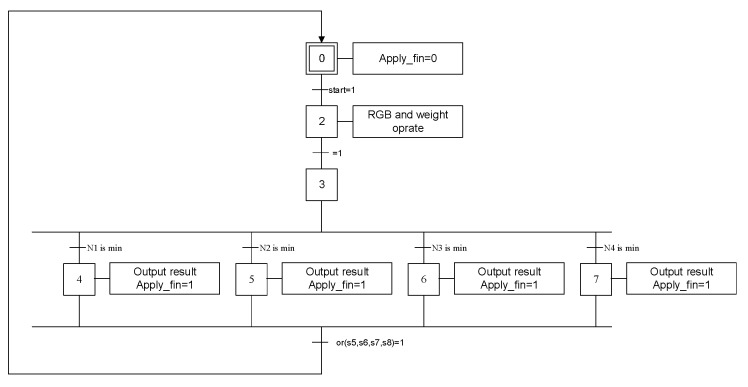
Discrete-event model of the SOM color classification module.

The SOM color classification module can be divided into two submodules: a distance calculation module and a decision module. These modules are mainly used for calculating input vectors and the distance of the weights for all output neurons, selecting the winner neuron, and determining the category of the input vector. Figure 9 and Figure 10 display the system architecture and the Grafcet discrete-event model of the SOM color classification module, respectively.

**Figure 11:**
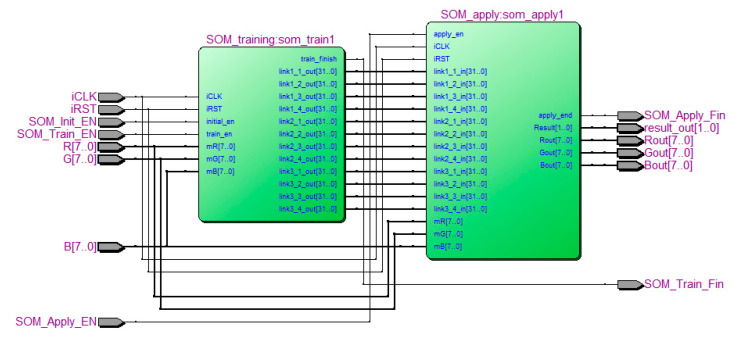
RTL-schematic diagram of the SOM-based image segmentation module.

Figure 11 shows the RTL-schematic diagram of the SOM-based image segmentation module, with the SOM training module on the left and the SOM color classification module on the right. According to the analysis results of the maximum clock frequency, the employed SOM-based image segmentation module processes 322 color images with a resolution of 640 × 480 per second. However, it can only process up to 50 color images with a resolution of 640 × 480 per second when coupled with the dual camera vision module, whose system clock constrains the processing speed.

#### 2.2.3. Connected Component Labeling Module

**Figure 12:**
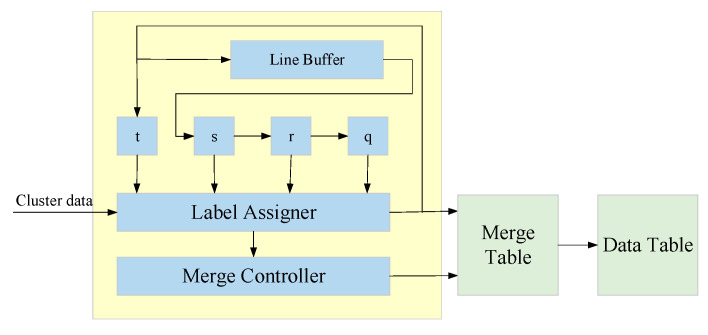
Hardware architecture of the CCL module.

Binary images are scanned in the CCL module through a raster scan, which is performed along a path from left to right and from top to bottom. Notably, the scanning of a pixel requires only identifying the position of its neighboring pixels. Figure 12 illustrates the hardware architecture of the CCL module, which shows that the information of neighboring pixels *q*, *r*, *s*, and *t* in the 8-connected component is stored using a line buffer. The labeling information obtained from the label assigner module and merge controller module is stored with the pixel information in a merge table and data table.

##### Sum-of-Absolute-Difference-Based Stereo Matching Module

**Figure 13:**
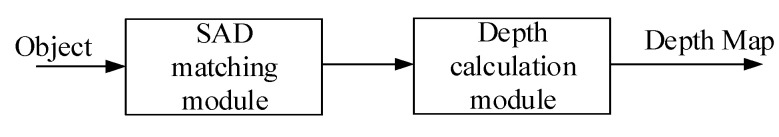
System architecture of the SAD-based stereo matching module.

The SAD-based stereo matching module constitutes two submodules: a SAD matching module and a depth calculation module. Figure 13 illustrates the system architecture of the SAD-base stereo matching module. The purpose of the SAD matching module is to perform the SAD matching computation for the left-image target blocks stored in the static random-access memory (SRAM) and the right images stored in the SDRAM. Then, the depth calculation module estimates depth information by applying a LUT to the obtained disparity values.

##### Pipeline Controller and System Integration

**Figure 14:**
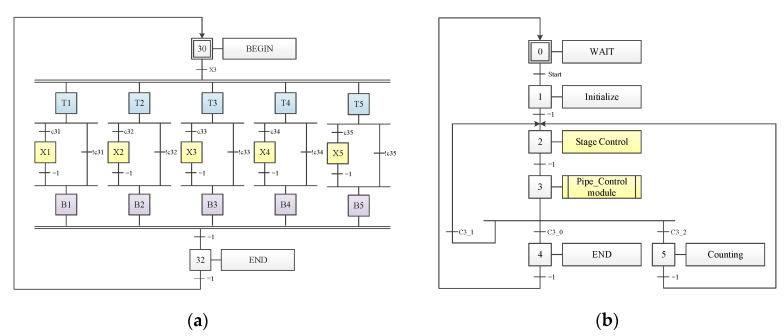
Discrete-event model of the pipeline controller. (**a**) Stage controller. (**b**) Top controller.

Pipeline techniques, which are widely used for designing controllers, improve controller performance by utilizing the parallel processing ability of hardware. In this study, a pipeline controller was developed to control the parallel processing procedures, the data flow, and communication among all modules. In Figure 14a, X1–X5 represent the primary operating modules of the proposed system, namely the dual camera vision module, SOM image segmentation module, CCL module, SAD-based stereo matching module, and video graphics array (VGA) controller, and T1–T5 and B1–B5 represent idle stages, which are used to stabilize the transfer of control signals. The pipeline controller views each circuit module as a task and conditionally controls them. On top of the pipeline controller, a top controller was designed to control state sequences (Figure 14b).

## 3. Results

### 3.1. Software Simulation and Verification

To verify the performance of the stereo vision algorithm, software simulation was conducted on a personal computer that had an Intel Core 2 E8400 3.00 GHz, 3-GB DDRIII RAM, and the 32-bit Windows 7 operating system. The software was developed using Borland C++ Builder 6. To compare the effects of image segmentation between various methods, an overlap measure was used as the criterion of evaluation. The formula of the overlap measure is given as [27]:

(8) $$overlap=\frac{TP}{TP+FP+FN^{’}}$$

where *TP*, *FP*, and *FN* denote the true positive (i.e., the area correctly classified as the object), false positive (i.e., the area incorrectly classified as the object), and false negative (i.e., the area incorrectly classified as the background) areas, respectively.

**Figure 15:**
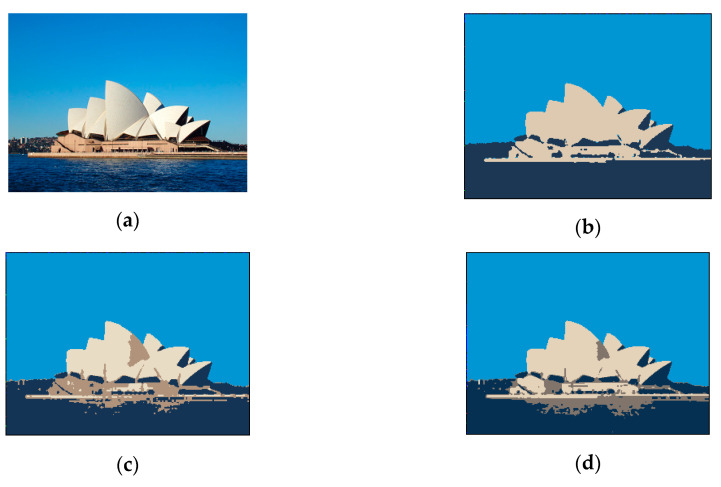
Segmentation results of an image of the Sydney Opera House. (**a**) Original image. (**b**) Ideal image segmentation. (**c**) Segmented image using K-means. (**d**) Segmented image using SOM.

Figure 15 shows the results of segmenting an image of the Sydney Opera House using different methods. Specifically, Figure 15a is the original image; Figure 15b is the ideal result of image segmentation; Figure 15c,d are images segmented using K-means and an SOM, respectively. A comparison of these images indicates that the SOM-based method generated more accurate results than did the K-means method. Table 1 presents a list of the results of applying these two methods to segmenting three different images, with the overlap measure and correct rate of both methods calculated.

**Figure 16:**
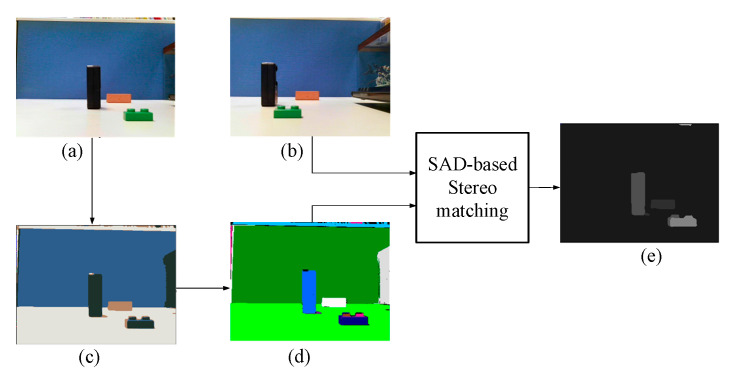
Results of the proposed stereo vision method. (**a**) Left image. (**b**) Right image. (**c**) Image after color classification. (**d**) Image after CCL. (**e**) Depth map.

**Figure 17:**
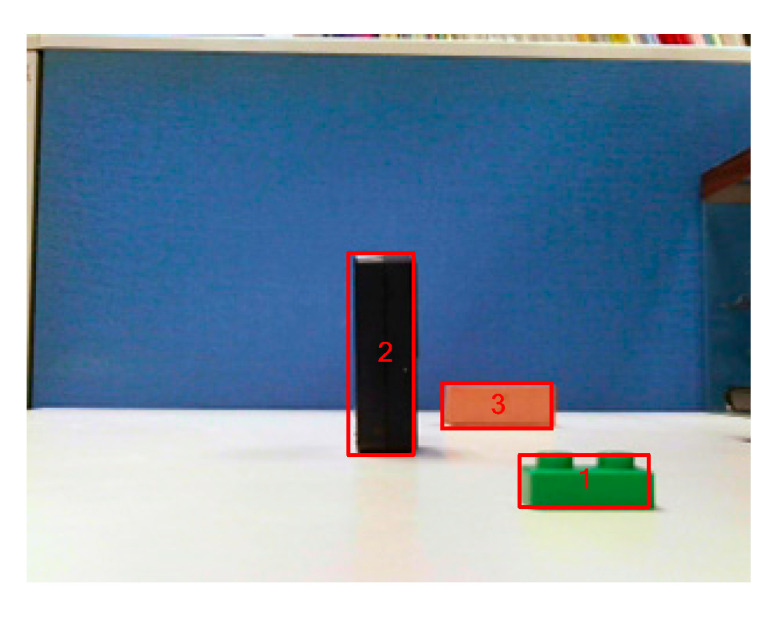
Labeled objects.

Next, a depth map was generated by applying CCL processing and stereo matching to the segmented objects (Figure 16). Figure 17 depicts the objects that were segmented, and the disparity values and estimated depth information derived from these objects (which suggest that the employed algorithm was able to accurately estimate depth information) are presented in Table 2.

### 3.2. Performance of the Embedded Stereo Vision System

**Figure 18:**
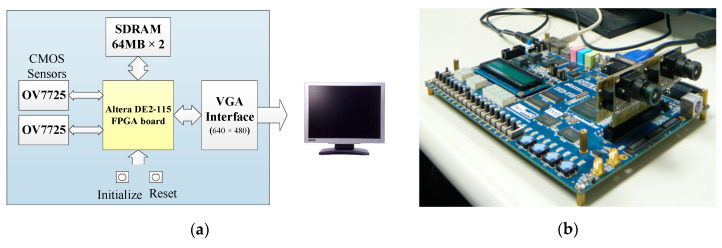
Hardware configuration of the embedded stereo vision system. (**a**) System hardware architecture. (**b**) Photo of the system.

The proposed embedded stereo vision system was implemented on an Altera DE2-115 FPGA board, which provides Altera Cyclone IV chips that enable the user to operate and verify their design. The system architecture and a photo of this architecture are presented in Figure 18a,b, respectively. During the operation process, a 128-MB SDRAM (with 32 MB of 32-bit memory) was used to store images and a 2-MB SRAM (with 1 MB of 16-bit memory) was used to store the information about the segmented object.

#### 3.2.1. System Performance Analysis

**Figure 19:**
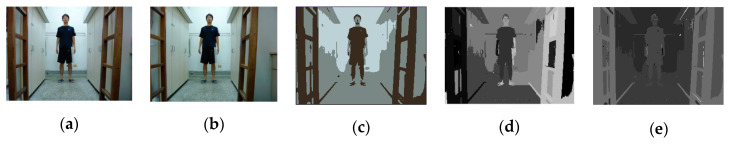
(**a**) Left image. (**b**) Right image. (**c**) Image after left-image object segmentation. (**d**) Image after left-image CCL processing. (**e**) Image after stereo matching.

A sample image was used to verify the performance of the proposed stereo vision system (Figure 19). Figure 19a,b are the left and right images captured by the dual camera vision module, respectively. Figure 19c,d present the results of object segmentation and CCL processing using the left image, respectively, and Figure 19e shows the results of the image after stereo matching and depth estimation were conducted. The image in Figure 19e confirms that the proposed system can accurately generate depth maps by utilizing left and right images.

#### 3.2.2. Hardware Resource Utilization and Performance Analysis

The resource allocation of the proposed stereo vision system is presented in Table 3. At a system frequency of 48 MHz, the dual camera vision module captured 50 color images with a resolution of 640 × 480 per second. The SOM training module has a system frequency of 6.63 MHz. By contrast, the SOM color classification module has a maximum system frequency of 105.88 MHz, and thus could immediately classify color images. A comparison of the performance of the existing SOM hardware accelerators when applied to process images with a resolution of 640 × 480 in terms of their learning and color classification rates was conducted. The results, presented in Table 4, indicate that the proposed system architecture produced higher learning and classification rates than did the other three previous methods, and therefore can perform real-time processing in an embedded system.

To further verify the improvement in the performance of all operating modules after they were implemented with hardware, an Altera DE2-115 control panel was used to load color images with a resolution of 640 × 480 into the SDRAM. The SDRAM controller was then employed to read the obtained image data and implement various image processing experiments. Next, the data derived from these experiments were compared with those obtained from software simulation. The comparison results, shown in Table 5, reveal that the proposed operation modules, after being implemented with hardware, had excellent performance, and exhibited higher computational efficiency than that of the simulation software program. In addition, the parallel operations and pipeline architecture effectively reduced the idle time of the circuit modules. The proposed stereo vision system can process 13.8 images with a resolution of 640 × 480 per second, which satisfies the requirement of real-time processing.

In this section, we show that the SOM method used in this research has good performance in several other methods in terms of learning rate and classification rate, and we also compare it with the software implementation of a computer with a system frequency of 3 GHz. Still can have outstanding performance. Besides, we are even better than the methods of Jin’s [28], Michalik’s [29], and Wang’s [30] in the use of FPGA resources, and also have quite excellent performance at a system clock of only 48 MHz.

## 4. Conclusions

Deep learning is the most popular method in contemporary computer vision research, but it requires a lot of system resources and power consumption. The verification platform used in this study is low power consumption, low cost, and low system clock. Even so, the method of this research has quite good performance in several aspects.

This study designed an SOM neural network module architecture and developed it into a hardware accelerator that effectively enhances the computational power of conventional modules in image segmentation. In short, neural network training and color classification can be efficiently performed using the proposed system. During the operation process, a pipeline architecture coupled with parallel processing functions was designed by incorporating the aforementioned SOM hardware accelerator, a dual camera vision module, a CCL module, and a stereo matching module into one stereo vision system that was assembled on an Altera DE2-115 board. Empirical verification determined that the proposed stereo vision system can process 13.8 color images with a resolution of 640 × 480 per second, and therefore has commercial value as a potential industrial application. 

## Figures and Tables

**Table 1:** Comparison of k-means clustering and the SOM neural network.

Method	Image	Opera House	Sky	Ocean	Total	Correct Rate
K-Means	TP	7058	38,400	15,994	61,452	0.9104
	FN	0	26	0	26	
	FP	3416	131	1455	5002	
	overlap	0.673	0.995	0.916	-	-
SOM	TP	9096	38,420	14,280	61796	0.9154
	FN	12	16	0	28	
	FP	1378	111	3169	4658	
	overlap	0.867	0.996	0.818	-	-
	Image size: 300 × 255					

**Table 2:** Estimated depth information.

Objects	Center-Point of Original Object	Center-Point of Target Block	Disparity	Estimated Depth	Observed Depth
1	(247,197)	(116,197)	131	18.8 cm	19.0 cm
2	(158,142)	(76,142)	82	36.2 cm	35.0 cm
3	(207,162)	(153,162)	54	56.0 cm	56.0 cm

**Table 3:** Resource allocation of the proposed stereo vision system on an altera cyclon IV chip.

	Module	Dual Camera Vision System	SDRAM	SOM-Based Image Segmentation	VGA Controller
Resources					
System clock		48 MHz	122.55 MHz	6.63 MHz–105.88 MHz	265.82 MHz
Total Logic Element		2117/114,480 (2%)	1672/114,480 (1%)	22,498/114,480 (20%)	79/114,480 (<1%)
Total Register		1429	757	2709	56
Total Memory Bits		425,952/3,981,312 (11%)	49,152/3,981,312 (1%)	0/3,981,312 (0%)	0/3,981,312

**Table 4:** Performance comparison with exiting SOM hardware architectures.

		Manolakos’s Method [22]	Kurdthongmee’s Method [20]	Porrmann’s Method [21]	Our Method
SOM Training Module	learning rate (vectors/s)	68,900	N/C	94,000	413,125
	system clock	148 MHz	24.2 MHz	40 MHz	6.63 MHz
SOM Color Classification Module	classification rate (vectors/s)	144,000	N/C	250,000	42,265,000
	system clock	148 MHz	24.2 MHz	40 MHz	105.88 MHz

**Table 5:** Comparison of time needed to process a 640 × 480 color image using a software program or hardware module.

	Software Program	Pipelined Hardware Module
Clock Frequency	3 GHz	84.53 MHz
Object Segmentation	0.039 s	0.004 s
CCL	0.037 s	0.066 s
Stereo Matching	0.122 s	0.064 s
